# Uncovering Networks from Genome-Wide Association Studies via Circular Genomic Permutation

**DOI:** 10.1534/g3.112.002618

**Published:** 2012-09-01

**Authors:** Claudia P. Cabrera, Pau Navarro, Jennifer E. Huffman, Alan F. Wright, Caroline Hayward, Harry Campbell, James F. Wilson, Igor Rudan, Nicholas D. Hastie, Veronique Vitart, Chris S. Haley

**Affiliations:** * MRC Human Genetics Unit, Medical Research Council, Institute of Genetics and Molecular Medicine, University of Edinburgh, Edinburgh EH4 2XU, Scotland, United Kingdom; † Centre for Population Health Sciences, University of Edinburgh, Edinburgh EH8 9AG, Scotland, United Kingdom; ‡ The Roslin Institute and R(D)SVS, University of Edinburgh, Easter Bush EH25 9RG, Scotland, United Kingdom

**Keywords:** GWAS, pathway-based, permutation method, genomicper R package, cardiac disease

## Abstract

Genome-wide association studies (GWAS) aim to detect single nucleotide polymorphisms (SNP) associated with trait variation. However, due to the large number of tests, standard analysis techniques impose highly stringent significance thresholds, leaving potentially associated SNPs undetected, and much of the trait genetic variation unexplained. Pathway- and network-based methodologies applied to GWAS aim to detect associations missed by standard single-marker approaches. The complex and non-random architecture of the genome makes it a challenge to derive an appropriate testing framework for such methodologies. We developed a rapid and simple permutation approach that uses GWAS SNP association results to establish the significance of pathway associations while accounting for the linkage disequilibrium structure of SNPs and the clustering of functionally related elements in the genome. All SNPs used in the GWAS are placed in a “circular genome” according to their location. Then the complete set of SNP association *P* values are permuted by rotation with respect to the genomic locations of the SNPs. Once these “simulated” *P* values are assigned, the joint gene *P* values are calculated using Fisher’s combination test, and the association of pathways is tested using the hypergeometric test. The circular genomic permutation approach was applied to a human genome-wide association dataset. The data consists of 719 individuals from the ORCADES study genotyped for ∼300,000 SNPs and measured for 51 traits ranging from physical to biochemical measurements. KEGG pathways (n = 225) were used as the sets of pathways to be tested. Our results demonstrate that the circular genomic permutations provide robust association *P* values. The non-permuted hypergeometric analysis generates ∼1400 pathway-trait combination results with an association *P* value more significant than *P* ≤ 0.05, whereas applying circular genomic permutation reduces the number of significant results to a more credible 40% of that value. The circular permutation software (“genomicper”) is available as an R package at http://cran.r-project.org/.

Genome-wide association studies (GWAS) have successfully identified many loci associated with complex traits and diseases (Wellcome Trust Case Control Consortium 2007; Hindorff *et al.* 2009). However, the identified single nucleotide polymorphisms (SNP) passing the highly stringent significance thresholds set in these studies explain only a small proportion of the traits’ variation (Manolio *et al.* 2009). Investigating variants of modest size effects using a gene-set analysis approach has been proposed to identify some of the undetected variation. Gene-set methods aim to identify effects of groups of genes, which may not be individually significant but, when analyzed jointly, may have a detectable effect on the phenotype or disease of the organism under study (Wang *et al.* 2007).

Most of the gene-set methodologies use random permutations to assess the statistical significance of the results. Typically, to generate a null distribution of pathway or gene-set association results, SNP or gene association *P* values are randomized post-GWAS [*e.g.*, gene ontology analysis ALLIGATOR performs gene resampling (Holmans *et al.* 2009), and i-GSEA4GWAS performs random SNP permutations (Zhang *et al.* 2010)] or, alternatively, phenotypic labels are randomized prior to GWAS [*e.g*,. SNP ratio test (O’Dushlaine *et al.* 2009) and RS-SNP random-set (D’Addabbo *et al.* 2011)]. However, SNP and gene-level permutations do not take into account the genomic structure, such as regional linkage disequilibrium (LD) and functional clustering of genes, effectively simulating a genome in which adjacent SNPs are uncorrelated by LD and genes are distributed randomly with respect to each other. Permutation approaches that ignore clustering generated by LD and functional co-location could create inappropriate test statistic null distributions. The possibility of performing case/control label permutations or other permutations prior to performing GWAS is limited as it requires the raw data which is often not available, and also given that each permutation is followed by the association analysis (GWAS) and subsequently with the gene-set testing, this results in a computationally expensive approach (Wang *et al.* 2011a). Furthermore, concern on the application and interpretation of gene-set–based methodologies has being raised and discussed previously (Wang *et al.* 2010, 2011b; Fridley and Biernacka 2011). Despite the lack of consensus on the most appropriate methodology and the warnings raised, GWAS gene-set approaches have being applied to diseases, such as breast cancer (Menashe *et al.* 2010), Crohn’s disease (Ballard *et al.* 2010), multiple sclerosis (Baranzini *et al.* 2009), and schizophrenia (Jia *et al.* 2010).

We have developed a permutation approach that uses GWAS results (single SNP association *P* values) to establish the significance of gene sets and pathway associations. The objective was to develop a method that would increase our understanding of the pathways involved in various traits and to detect the effects that would have been missed by traditional single-marker analysis without generating an excess of false-positive pathway associations. We explore the performance of pathway-based approaches applied to GWAS using this new “circular genomic permutation” approach. This approach is equivalent to the simulation of pathways with the same number of genes as the pathway under study and with a structure that faithfully reflects that found in the genome with regard to conservation of LD and relative location of features (SNPs and genes), but with the SNP effects on the phenotype randomized and no real pathway effects. This allows us to obtain appropriate distributions of the test statistics under the null hypothesis of no association for every pathway/trait combination surveyed. We compare our approach with that of applying the hypergeometric test alone, which assumes that the null distribution is hypergeometric, or of generating a null distribution by random permutations at the SNP and the gene levels, which does not take full account of genomic architecture. In addition, we attempt to measure the variation explained by the SNPs acting under a pathway.

## MATERIALS AND METHODS

### Genome-wide association data and analysis

The association values were obtained from the Orkney Complex Disease Study (ORCADES) (McQuillan *et al.* 2008).The cohort consisted of 719 individuals measured for 51 traits ranging from physical to biochemical measurements. These traits include blood pressure, height, waist circumference measurements, and cortisol and cholesterol levels, among others (for full descriptions, see Table S1). Individuals were genotyped using the Illumina HumanHap 300v2 array (which contains in excess of 300,000 SNP markers; http://www.illumina.com/). The SNP association *P* values were calculated for each trait as described here. Each trait was adjusted for sex and age, and the residuals were transformed to ensure their normal distribution using quantile normalization. The mixed linear model mmscore() function of the GenABEL package (Aulchenko *et al.* 2007) for R statistical software (http://www.R-project.org) was used to test association between SNP and trait under an additive model for the SNP fixed effect. This score test for family-based association takes into account pedigree structure and allows unbiased estimations of SNP allelic effects when relatedness is present between individuals in the population sample (Chen and Abecasis 2007). The relationship matrix used in this analysis was generated by the ibs() function of GenABEL (using weight = “freq” option), which utilizes IBS genotype sharing to estimate the realized pairwise kinship coefficient.

### SNP annotations

The genomic location of every SNP in the dataset was updated using the WGAViewer software annotation tool (Ge *et al.* 2008). Gene annotations (*i.e.*, gene identifiers, symbols, and locations) were extracted from the NCBI Gene database (http://www.ncbi.nlm.nih.gov/gene; build.37.1). The SNP-to-gene annotation was performed by linking the SNP genomic location and the gene genomic locations. Two annotation datasets were created according to the physical distance of the SNPs to the genes. “Distance 0” refers to the dataset where the SNPs were annotated to a gene if they were located within the start and end of transcription of the gene (including SNPs falling within the introns of a gene). The second annotation annotated SNPs to genes if they were within a 20 kb distance of the gene(s), also from the start to end of transcription. SNPs from both datasets may be annotated to more than one gene, and many SNPs do not annotate to any gene.

### Pathway analysis: gene *P* values and hypergeometric test

KEGG pathways were used as the predefined gene sets. The significance of the association of a pathway with each trait was evaluated using the hypergeometric test. Under the assumption of independent variables, the hypergeometric test gives the probability of having *x* number of “significant genes” belonging to the same pathway.

$$P=1-\sum_{i=0}^{K}\frac{\left(\begin{array}{c} S\\ i \end{array}\right)\;\left(\begin{array}{c} N-S\\ m-i \end{array}\right)}{\left(\begin{array}{c} N\\ m \end{array}\right)}$$

where *m* represents the number of genes in the pathway, *K* is the number of significant genes in the pathway, *N* the total number of genes contained in the dataset, and *S* is the total number of significant genes in the dataset (according to their joint gene *P* values). To apply the hypergeometric test, we needed one *P* value per gene; however, the number of SNPs mapped to a specific gene varies across the whole dataset, and therefore, a variable number of SNP *P* values of association are assigned to each gene. The joint gene *P* value was calculated using the Fisher’s combination test as described by Peng *et al.* (2010).

$$Z_{F}=-2\sum_{i=0}^{K}\log\;P_{i}$$

where *P_i_* represents the SNP association *P* values, and *K* is the number of SNPs to be combined.

For this study, we considered the genes to be significantly associated with a trait if their gene *P* value is less than α ≤ 0.05. The hypergeometric test was calculated for the 51 traits and for each of the 225 pathways in the KEGG database (Ogata *et al.* 1999).

### Circular and random permutations

We used circular genomic permutations, gene-level random permutations, and SNP-level random permutations to obtain the distributions of the hypergeometric test pathway-association *P* values under the null hypothesis of no association. The three permutation types were performed for each test (for each pathway for each trait) and are described below.

#### Circular genomic permutations:

Our methodology performs the permutations at the SNP-level in a genomic manner. The complete set of SNP association *P* values are ordered according to their genomic position, first by chromosome and then by their location in the chromosome, including those SNPs genotyped in our data but not annotated to any gene. We consider the genome to be circular and ordered from chromosome 1 to chromosome X and restarting at chromosome 1 again. Then the complete set of SNP association *P* values are permuted by rotation with respect to their genomic locations, *i.e.*, a random number between 1 and the total number of SNPs is drawn, and the *P* value associated with the first SNP in the genome rotates to that of the random number-th SNP and all other *P* values rotate to the same degree to the corresponding SNPs. SNPs thus retain the same position with respect to each other but, at each permutation, gain new random values of association with adjacent *P* values showing similar patterns of correlation as found in the original data. For each permutation, the joint gene *P* values are calculated using Fisher’s combination test followed by the hypergeometric test (Peng *et al.* 2010). This process is repeated 10,000 times to obtain the test statistic distribution under the null hypothesis of no pathway effect on the phenotype.

The pathway significance threshold was set at 5% of the distribution of the test statistics obtained from each pathway-trait test permutation; each pathway-trait test distribution consists of 10,000 permuted hypergeometric results. The hypergeometric-empirical *P* values were calculated from the ranked position of the hypergeometric-theoretical *P* value in the permutations.

#### Gene-level and SNP-level random permutations:

To explore how circular permutations perform compared with random permutations, alternative permutation strategies were applied to the distance 0 dataset. These permutations randomly attributed the observed trait-association *P* values to either SNPs or genes, as appropriate, without accounting for spatial (and other possible) correlations among them. Both random permutation procedures consisted of 10,000 runs on every trait for all the 225 pathways.

##### SNP-level random permutations:

The difference between the SNP-level random permutation and circular permutation is that the association *P* values are randomly given to *n* SNPs, where *n* is the total number of SNPs per gene for each trait in the initial GWAS. This simulates a situation where SNPs within a gene are not correlated due either to LD or to their collocation within a gene. Random selection of SNP *P* values is followed by the Fisher’s combination test and the hypergeometric test.

##### Gene-level random permutations:

For the gene-level random permutations, the gene *P* values where calculated once for the original (non-permuted) dataset. This permutation consists of re-sampling genes according to the number of genes represented in our dataset per pathway with their *P* values. This simulates a situation where genes within a pathway are not correlated due to any spatial or functional clustering within the genome.

## RESULTS

### GWAS data annotations

After quality control and genome-wide analysis of the data, 318,235 SNP association *P* values were taken for further analyses. Approximately 42% of the SNPs were successfully annotated to a gene (∼137,808 SNPs) at distance 0. The percentage of annotated SNPs rises to ∼61% for the 20 kb annotation dataset. According to the NCBI gene statistics, currently there are ∼42,059 unique human gene identifiers (as of February 2012; http://www.ncbi.nlm.nih.gov/projects/Gene/gentrez_stats.cgi?TAXORG=9606). The numbers of unique gene identifiers observed in the datasets were 17,806 and 30,580 for distances 0 and 20 kb, respectively. The analyzed pathways have a total of 5859 unique genes. We found 4403 genes mapping to pathways in the distance 0 dataset, whereas in the 20 kb dataset, the number goes up to 5595 genes.

### Hypergeometric test results

For each dataset, 11,475 hypergeometric tests were performed (from a combination of 225 pathways and 51 traits). At a *P* ≤ 0.05 threshold, we would expect ∼574 significant results by chance alone. The hypergeometric results of the distance 0 dataset produced 1423 tests below the 0.05 threshold; therefore, more than twice the number of significant tests than expected by chance was observed. The number of significant tests below the 0.05 threshold on the 20 kb annotation increased to 1805.

### Circular genomic permutation results

#### Distance 0:

**Figure 1:**
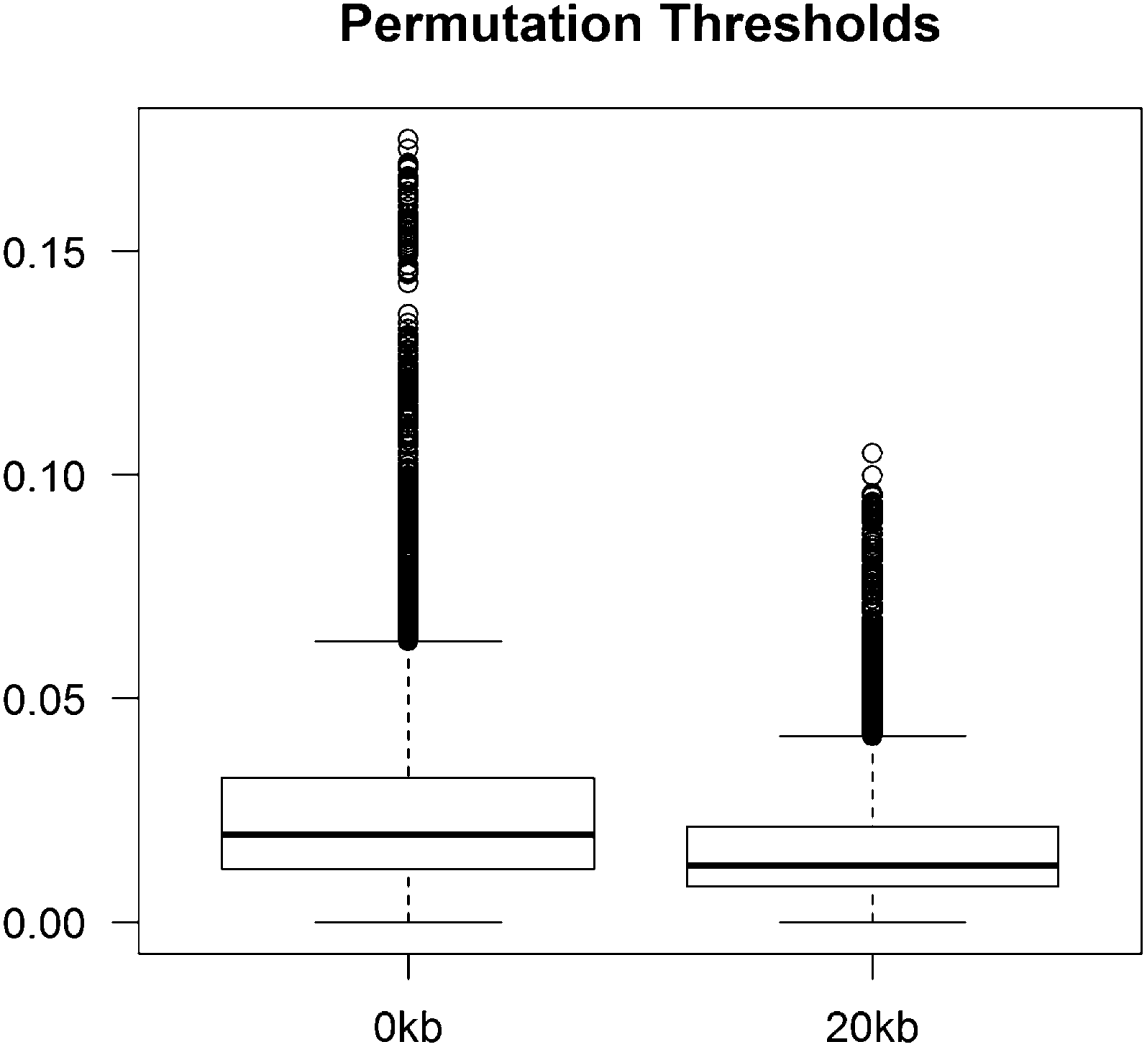
Threshold distributions at distances 0 and 20 kb. Distance 0: The first quartile of the tests sets a threshold of ∼0.02 and the third quartile is set at ∼0.03, whereas the maximum value was equal to 0.175. The range of threshold distributions at 20 kb was smaller than that observed at distance 0. The first quartile had a threshold of ∼ 0.008, the third only went up to ∼0.02, and the maximum threshold set was 0.093.

The results based on the empirical pathway significance threshold found 536 pathway-trait tests to be statistically significant (Table S2). The range of the pathway significance thresholds observed across all the tests (all pathway-trait combinations) was very variable (Figure 1). For distance 0, the maximum empirical pathway significance threshold across tests corresponded to a *P* value of 0.175 (*i.e.*, for one pathway-trait combination, hypergeometric-empirical *P* values below 0.175 would be significant at the 0.05 level as determined empirically by circular permutation). However, for most of the pathway-trait combinations, the hypergeometric-theoretical *P* value of 0.05 was too liberal for the empirical threshold of 0.05. For the empirical *P* value threshold of 0.05, the inter-quartile distribution (*i.e.*, central 50% of the tests) corresponded to *P* values from ∼0.02 to ∼0.03. As a result, more than 60% of the hypergeometric tests that were classified as significant when using the hypergeometric test alone do not remain statistically significant at α (*P* ≤ 0.05) if the empirical pathway significance thresholds are used. The opposite was also observed for some trait-pathway combinations; thus, five results with the hypergeometric-theoretical *P* value less significant than α (*i.e.*, non-significant), are deemed to be significant according to the permutations. These results correspond to four different pathways and five different traits.

#### Distance 20 kb:

A total of 594 tests were found to be significant at the empirical threshold (Table S3). The pathway significance thresholds were smaller than those observed at distance 0. The inter-quartile threshold range was from ∼0.008 up to ∼0.02, and the maximum pathway significance threshold set was equal to 0.093 (Figure 1). Here four tests were also observed to be significant in the permutations but with a theoretical hypergeometric probability less significant than α (*P* ≥ 0.05). However, these tests are not the same as the ones detected by the distance 0 analysis. The analyses at distances 0 and 20 kb share a total of 198 (∼34%) significant tests (Table S4).

### Hypergeometric test *vs.* circular permutations

**Figure 2:**
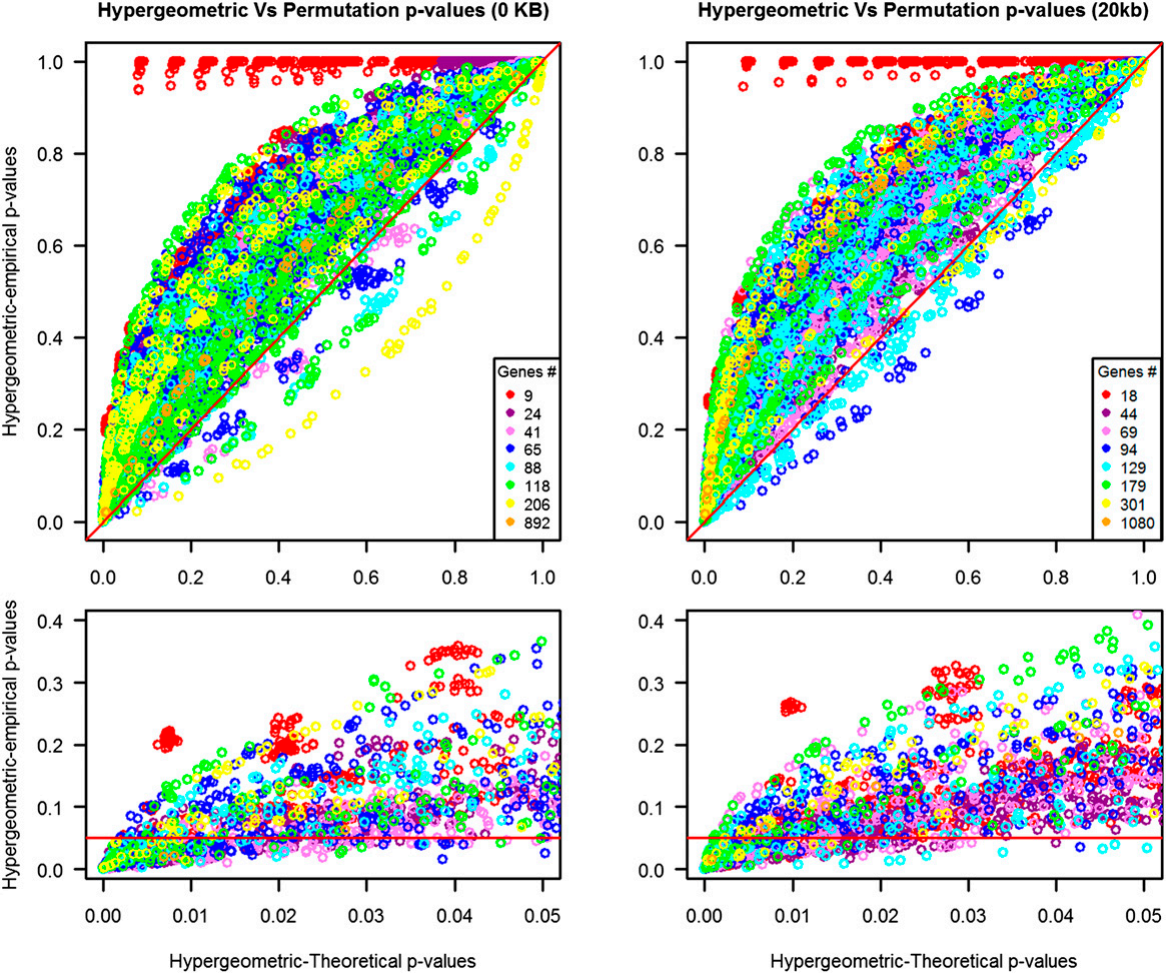
Hypergeometric-theoretical *P* values *vs.* hypergeometric-empirical *P* values at distances 0 and 20 kb. Visual representation of the relationship of the hypergeometric-theoretical *P* values (x-axis) compared with the hypergeometric-empirical *P* values from the permutations (y-axis). To assess the effect of the size of the pathways (*i.e.*, number of genes in the pathway), *P* values are colored by pathway size. For this representation, pathways were clustered using k-means with eight groups. The legend represents the centers of that size group. (Top plots) The red line represents the trend that would be followed if the hypergeometric-empirical *P* values would match perfectly to those of the hypergeometric-theoretical *P* values. (Bottom plots) Close-up from the top plot, where the red lines are fixed at a 0.05 empirical threshold. The close-up graph represents all the tests below an arbitrary threshold set at 0.05 when using the hypergeometric test alone. The results below the line represent the significant results when applying circular permutations; results above the line are those which were not longer significant according to this method.

The hypergeometric-theoretical *P* values were compared with those obtained by permutation analysis. The correlation between the hypergeometric-theoretical *P* values and the hypergeometric-empirical *P* values seemed to be very high (*i.e.*, 0.840 and 0.885 for the 0 kb and 20 kb analyses, respectively). The hypergeometric-empirical *P* values deviate notably from the estimated probability of the hypergeometric test; for most tests, the hypergeometric-empirical *P* values are larger (*i.e.*, less significant) than those obtained by the hypergeometric test (Figure 2).

To investigate further the causes of the difference between the hypergeometric-theoretical *P* values and the permutation results, we carried out linear regressions in which our observed variables were the hypergeometric-theoretical *P* values, the hypergeometric-empirical *P* values, and the difference between the hypergeometric-theoretical *P* values and the hypergeometric-empirical *P* values. The predictive variables used were functions of the number of genes, the number of SNPs, or the SNP-gene ratio in the pathways. These predictors were categorized into groups representing the size of the pathway (*i.e.*, amount of genes/SNPs/SNP-gene ratio on each pathway). For example, pathways containing from 1 to 25 genes would belong to category A, while pathways containing 26–50 genes would belong to category B. The pathways analyzed consisted approximately of 100 different pathway sizes according to the number of genes, ∼200 according to the SNPs, and ∼130 according to the SNP-gene ratio. To assign the pathways to their representative category, we used the *k-means* algorithm (Hartigan and Wong 1979) within R. Genes were grouped in 5 clusters, SNPs in 12, and the SNP-gene ratio into 7. The *r^2^* results demonstrate that none of these three predictive variables has a strong impact on the theoretical or the empirical results (Table 1). However, a high *r^2^* was observed for the regression on the differences between theoretical and the empirical *P* values, where the *r^2^* goes up to 0.431 for the number of SNPs being the predictive variable, where the difference between the methodologies is greater in pathways of extreme sizes (*i.e.*, very small pathways containing ∼23 SNPs or very large pathways ∼1,500 SNPs), implying that the number of SNPs in the pathways may influence the differences between the two methodologies. However, this was not surprising as the *P* values vary widely between the theoretical and the empirical approaches, especially for those extreme-sized pathways where more significant results (small *P* values) were observed by the theoretical-hypergeometric test, but when analyzed through permutations, these results obtain very large *P* values.

**Table 1:** *r*^2^ results for hypergeometric-theoretical *P* values, hypergeometric-empirical *P* values, and *r*^2^ difference between the hypergeometric-theoretical *P* values and the hypergeometric-empirical *P* values

	Hypergeometric *r*^2^	Empirical *r*^2^	Difference *r*^2^
Genes	0.014	0.039	0.225
SNPs	0.061	0.058	0.431
SNP-gene ratio	0.056	0.013	0.082

The predictive variables used were functions of the number of genes, the number of SNPs, or the SNP-gene ratio in the pathways. These predictors were categorized into groups representing the size of the pathway (*i.e.*, amount of genes/SNPs/SNP-gene ratio on each pathway).

#### Random permutations vs. circular permutations:

**Figure 3:**
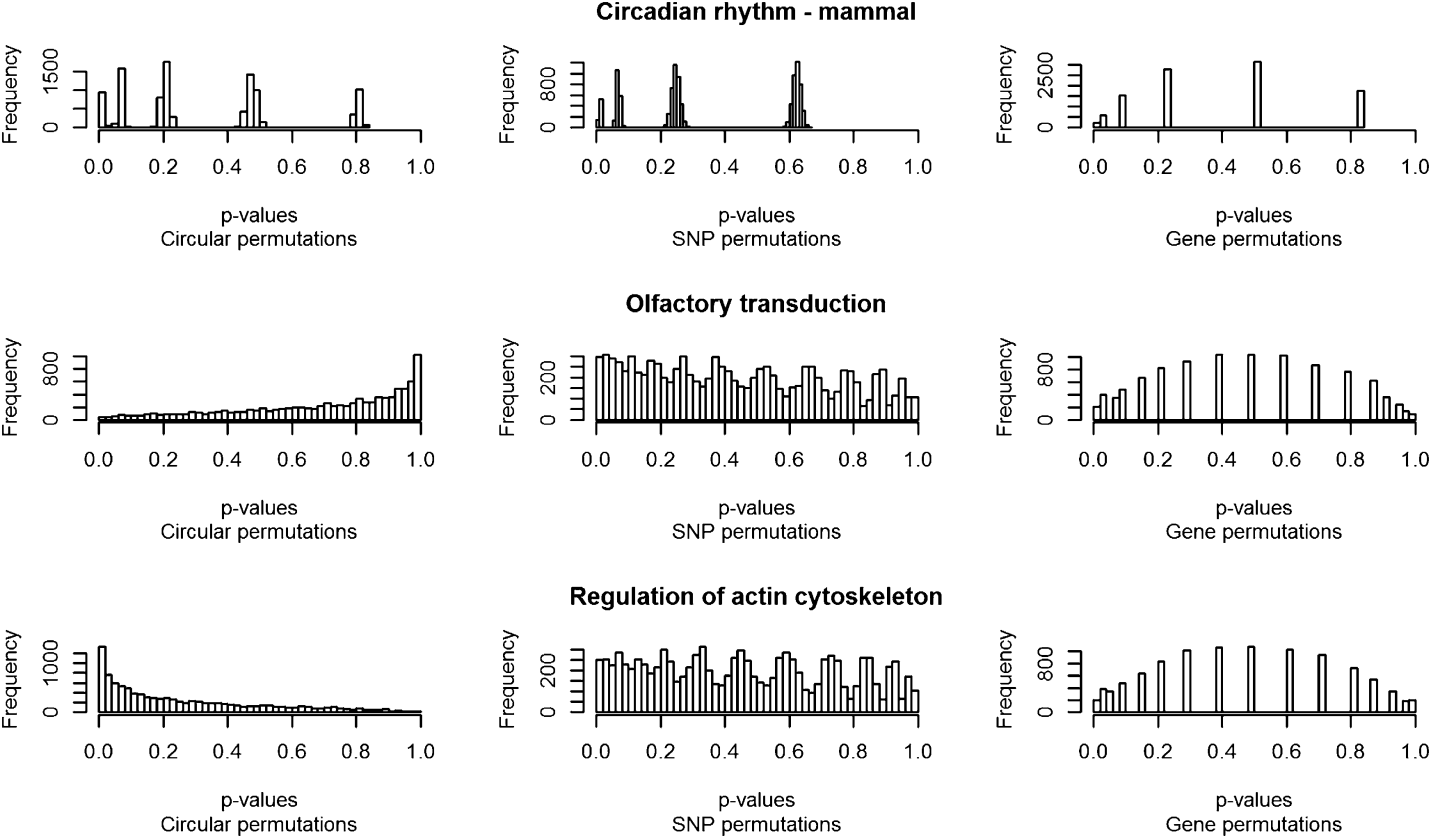
Permutation distributions. Three pathways and their hypergeometric *P* value permuted distributions for the glucose trait. Each individual plot represents the outcome of the 10,000 permuted hypergeometric tests per pathway. These three pathways were selected because they represent the three trends observed across all the analyzed pathways. The left column represents the circular genomic permutation *P* value distribution; the central column represents the SNP-level random permutation *P* value distribution, and the right column represents the gene-level random permutation *P* value distribution.

We explored the distribution of individual tests of the permuted hypergeometric results (*i.e.*, distribution of *P* values for each pathway-trait combination for both random permutations and the circular permutations). Figure 3 shows these distributions for the glucose trait across three pathways and highlights the differences between them across permutation methods and pathways. Each plot represents the outcome of the 10,000 permuted hypergeometric tests.

**Figure 4:**
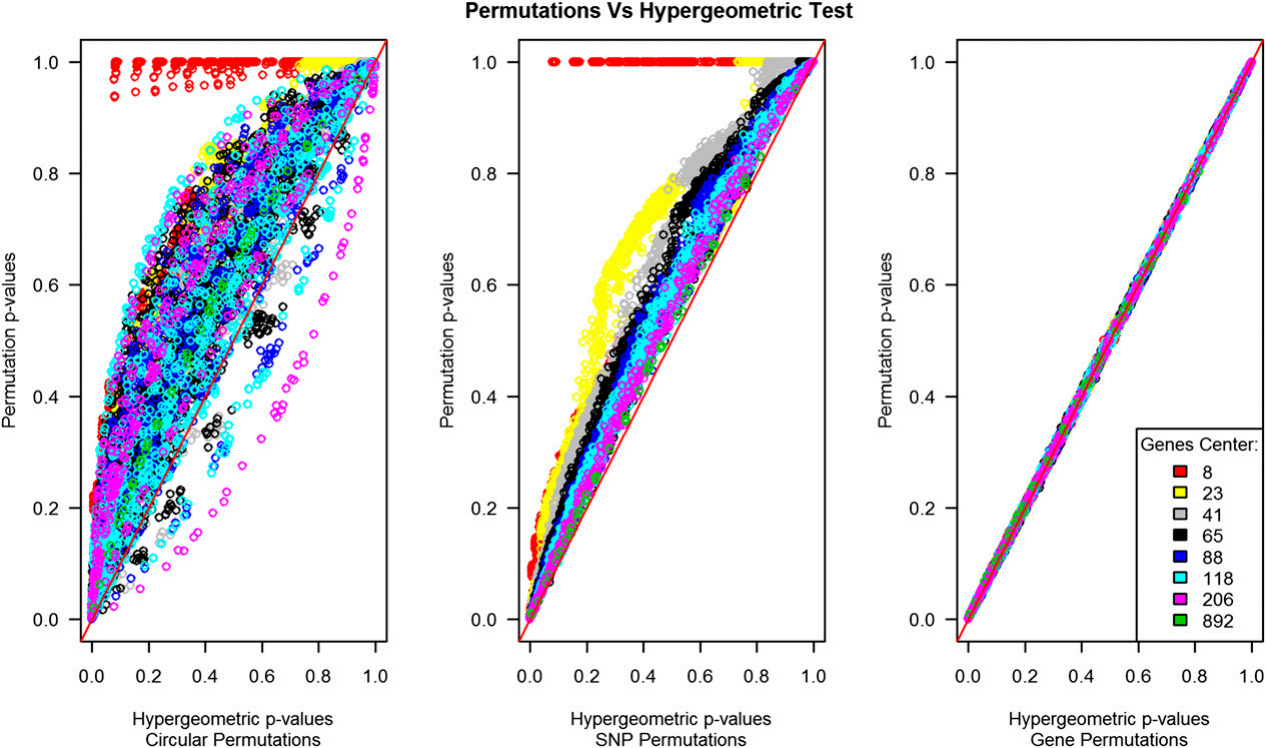
Permutation methods. This plot compares the hypergeometric *P* values on the x-axis to the permutation *P* values in the y-axis. The three permutation methodologies are represented (circular genomic permutation, the SNP-level random permutation, and the gene-level random permutations).

In Figure 4, we can observe the hypergeometric-empirical *P* values obtained through the various permutation methodologies compared with the hypergeometric-theoretical *P* values. Here, gene-level random permutation results seem almost identical to those predicted by the hypergeometric test. The correlation between the hypergeometric-theoretical *P* values and the hypergeometric-empirical *P* values for the gene-level random permutations was 0.99, and the maximum difference observed among all the 11,475 tests was only 0.02. This degree of similarity is not observed in the SNP-level permutations (Figure 4).

### Variance explained by arrhythmogenic right ventricular cardiomyopathy: an example

**Figure 5:**
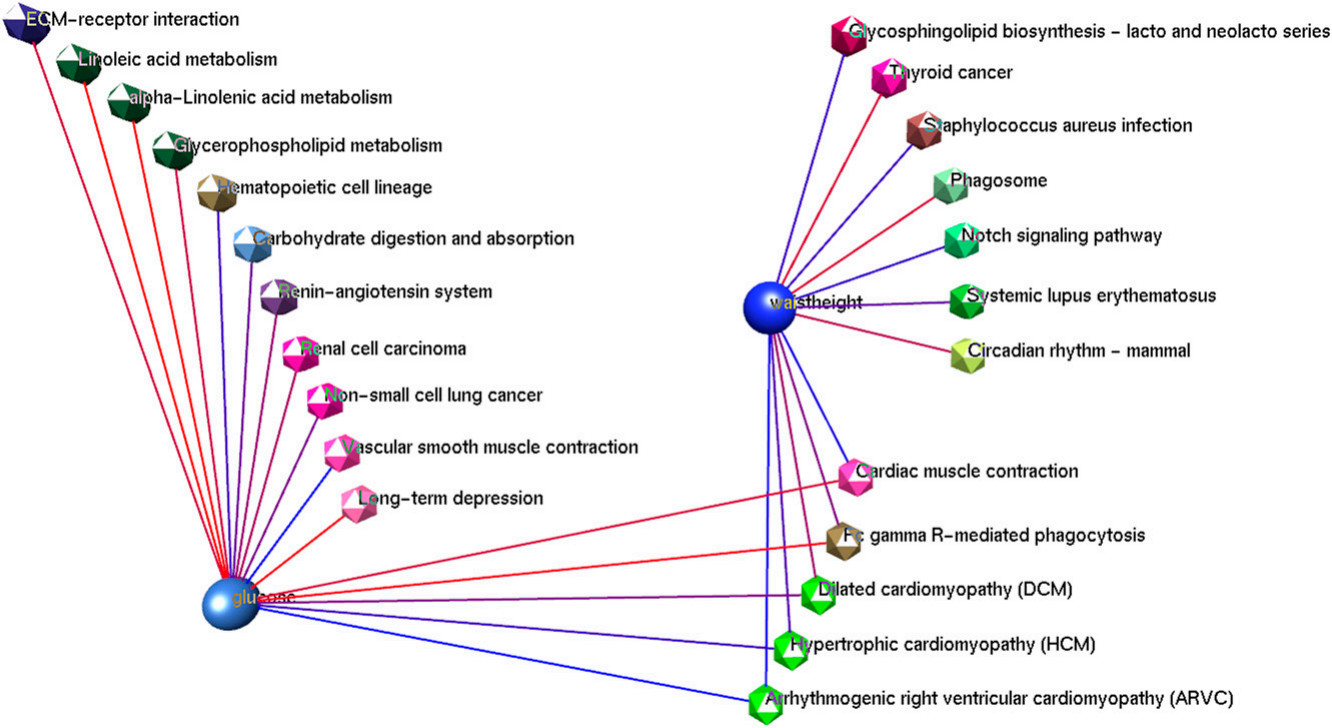
Significant tests related to the arrhythmogenic right ventricular cardiomyopathy (ARVC) pathway. Two traits (glucose and waist-to-height ratio) were found to be significant for the ARVC pathway. Both traits share a total of five pathways significant for both traits. Traits are represented by sphere nodes, and pathways are represented by the icosahedron nodes. The pathways are colored according to their pathway category. The link between the pathways and the traits (edges) represent the significant results obtained through the circular genomic permutation approach. Edges are colored from a blue-to-red scale, where blue represents the most significant results (*i.e.*, the ARVC pathway and the trait glucose hypergeometric-empirical *P* value = 0.0004, whereas the linoleic acid metabolism and glucose hypergeometric-empirical *P* value = 0.044). Image produced using BioLayout Express (Freeman *et al.* 2007).

To determine whether the pathway-based circular permutation approach detects more effects and can explain more variation within a trait than the traditional single-marker approach; the arrhythmogenic right ventricular cardiomyopathy (ARVC) pathway was selected to investigate how much variance was explained by the SNPs within the pathway. ARVC was selected because it was found to be highly significant in the distances 0 and 20 kb datasets for the trait glucose (hypergeometric-empirical *P* values of 0.0004 and 0.0006, respectively). The traits glucose and waist-to-height ratio were the only traits found to be significant for the ARVC pathway. Four other pathways were significant for the same two traits: cardiac muscle contraction, dilated cardiomyopathy (DCM), hypertrophic cardiomyopathy (HCM), and fc gamma R-mediated phagocytosis. Figure 5 is a representation of the significant pathways and traits related to ARVC. However, ARVC, HCM, and DCM share 50 genes in total. This represents 72% of the ARVC genes, 65% of HCM, and 63% DCM; there are no genes shared between the cardiovascular disease pathways and the fc-gamma R-mediated phagocytosis. The fc-gamma receptors are linked to the initiation of various signals (*e.g*., actin-cytoskeleton reorganization, and phagosomal membrane remodeling) (Garcia-Garcia and Rosales 2002).

The mixed linear model implemented in the *polygenic*() function of GenABEL was used to calculate the variance explained by the SNPs with an association *P* value more significant than α (*P* ≤ 0.05) for the trait glucose and all traits measured for the ARVC pathway. The analysis was performed using the distance 0 annotation. A total of ∼2311 SNPs were mapped to ARVC, from which 175 were below an association *P* value of 0.05 for the glucose trait. The percentage of the variance explained was given by the difference between the total estimated heritability and the estimated heritability with fixed effects, divided by the total estimated heritability. The estimated percentage of the variance of the trait glucose explained by the SNPs in the ARVC pathway with *P* value more significant than α ≤ 0.05 was ∼24%. In addition, only three other traits had some of the variance explained by the same SNPs: ∼20% variance of Hba1c (glycated hemoglobin, a measure of the average glucose plasma levels over time); ∼18% of CRP (C-reactive protein, an independent biomarker for cardiovascular diseases) (Kones 2010); and ∼40% of CIMT (carotid intima-media thickness, a measure of atherosclerosis). The phenotypic correlations of Hba1c, CRP, and CIMT with the trait glucose are 0.37, 0.03, and 0.10, respectively. No other trait had any significant amount of its variance explained by these SNPs in the ARVC pathway.

## DISCUSSION

In spite of the substantial developments in genetics and genomics, there are many unresolved questions, and the mechanisms driving complex diseases remain very poorly explained. Major advances have become available by sequencing genomes, characterizing their structure, and identifying the common elements across species (Mouse Genome Sequencing Consortium 2002). Despite these advances, there are still regions whose functions or mechanisms of action are still unknown or not fully understood; an example of this is a recent study that demonstrates natural selection also acting on non-coding regions (Mu *et al.* 2011). Furthermore, gene expression studies have identified chromosomal regions, groups of contiguous genes characterized by coordinated expression and similar transcriptional profiles (Caron *et al.* 2001; Versteeg *et al.* 2003). This has led to the development of approaches to take into account physical locations and genomic distances (Callegaro *et al.* 2006; Seifert *et al.* 2011).

Here we propose a novel approach for testing association of gene sets taking genomic structure and non-independence among SNPs into account: the circular permutation method.

The circular genomic permutation was applied to a small study (N = 719 individuals). Single-marker GWAS analysis detected 11 SNPs across three genomic regions and three traits more significant than 5 × 10^−8^ (generally accepted GWAS threshold). The small number of individuals genotyped in this study results in low power to detect associations reaching the stringent genome-wide threshold. This in turn results in true associations remaining undetected. The aim of the gene-set analysis using circular genomic permutation is to detect associations missed by standard GWAS single-marker association analyses by identifying SNPs with joint effects on a phenotype. Although our study provides a large number of phenotypic data, a larger study would have the potential to yield results that give us a better biological insight of the gene sets.

The methodology was applied to a study genotyped on a single platform. In instances where studies use a variety of platforms, imputation of genotypes at loci that are not present in all platforms, prior to the association analysis that will produce the association *P* values that are used in our gene-set analysis is recommended.

We included the X chromosome in the analysis (and permutations). LD in chromosome X is higher than for the autosomes (Schaffner 2004). We do not expect this to introduce a substantial bias in our case where the number of SNPs on the X chromosome is not high (∼2% of the total number of SNPs). However, an alternative approach could be to perform the permutations at a chromosomal instead of at the genomic level.

We compared the performance of our approach with that of two other, often used permutation methods (*i.e.*, gene-level and SNP-level random permutations). The hypergeometric-empirical *P* values of the gene-level random permutations agreed largely with the hypergeometric-theoretical *P* values because the number of significant genes is constant across all tests and the only source of variation is derived from how many of the re-sampled genes fall in pathways below the α threshold. Not all correlations of the initial SNP association *P* values are broken in this type of permutation, as the gene *P* values were derived from consecutive SNPs that were annotated to the same gene. In addition, the simulated pathways would mainly contain non-correlated gene *P* values, and therefore, the assumption of the hypergeometric test is not violated in this scenario. For the SNP-level random permutations, the SNPs are taken randomly from the genome and a new gene *P* value calculated from non-correlated SNPs, leading to different number of significant genes in the pathways and also in the universe (complete dataset), consequently generating more variation in the permuted hypergeometric results. In contrast, the circular genomic permutation, which is also performed at the SNP-level where the number of significant genes in the pathway and in the universe are not fixed (similarly to the SNP-level random permutations), seems to capture the genomic structure and shows how likely an outcome is expected according to this structure (Figure 3).

Finding a consensus between pathway-based approaches represents a challenge. Several methodologies demand a single association value per gene. Many of the methodologies select the strongest SNP signal of the gene, but doing so will overestimate the association for pathways with long genes. We chose to calculate a single gene *P* value through the Fisher’s combination test, although this may be conservative for the opposite reason—long genes with true associations may contain many SNPs with no functional effect that are not in LD with any functional variants. Another parameter to select is the window size to apply in the SNP-to-gene annotation. Based on the findings of Veyrieras *et al.* (2008), which estimated that 95% of the expression quantitative trait loci localize within the genes and within 20 kb from the genes, the 20 kb window-size annotation is becoming more popular (Holmans *et al.* 2009; Wang *et al.* 2011b). A clear advantage of including a window region for the annotation is the number of SNPs that will be annotated to genes. Here the number of SNPs successfully annotated to genes increased by 20% when the annotation window was set to 20 kb, allowing the capture of 1192 genes mapped to pathways that were missed before but that may influence the trait. An undesired effect may be the addition of noise to the analyses, as some SNPs included in the larger window-size analyses will have no real effect on the trait, and the proportion of SNPs annotated to various genes that might be acting under different pathways may increase. The effect of the noise on the data may also be observed on the differences between the pathway significance thresholds set through permutations, where the distance 0 analysis sets on average a pathway significance threshold of ∼0.02–0.03, and the distance 20 kb, ∼0.008–0.02. The distribution of the 20 kb permuted-hypergeometric *P* values approximates to the distributions observed in the gene-level random distributions. The overlap between the significant results of the distances 0 and the 20 kb datasets was weak; only 198 tests out of 536 (0 kb) and 594 (20 kb) were observed as significant in both analyses, suggesting that the annotation has a substantial contribution to the pathway association results. Some instances in which the distances 0 and 20 kb analyses agreed included two tests of the five in distance 0 found significant through permutations but with a theoretical hypergeometric *P* > 0.05. They were both confirmed as significant in the 20 kb analyses with a hypergeometric test *P* value of *P* = 0.04 and *P* = 0.001, and both also significant through the circular permutations of 20 kb.

In both analyses (distances 0 and 20 kb), the correlation of the hypergeometric-empirical *P* values with the hypergeometric-theoretical *P* values is very strong. However, the strong correlation only shows the degree of similarity between the ranked order between the empirical *P* values and the theoretical *P* values. As shown in Figure 2, the empirical *P* values tend to be larger than those obtained only through the hypergeometric test, which means that the use of the hypergeometric test alone will lead to false-positive results.

We also looked at the impact of the number of SNPs and the number of genes of the pathway. As shown in Figure 2, the hypergeometric-theoretical *P* values below 0.05 but not empirically significant at the same level included pathways of different sizes in terms of number of genes, and this pattern was also observed when the tests results were clustered by SNPs (Figure S1).

We presented as an example the significant pathways and traits linked to one of the most significant pathway-trait associations found through this methodological study. The ARVC pathway groups genes involved in one type of cardiomyopathy (characterized by replacement of the myocardium by fibrofatty tissue). Cardiomyopathy is a dysfunction of the heart muscle highly associated with sudden death, especially in the young (Towbin *et al.* 2006). No scientific references were found for the link between ARVC and the trait glucose. However, a close form of inherited cardiomyopathy has been linked to the PRKAG2 gene, a master regulator of glucose and lipid metabolism (Gollob *et al.* 2001), and one of the major causes of dilated cardiomyopathy, the most common type of cardiomyopathy, is hypertension and ischemic cardiomyopathy (Hunt *et al.* 2005). The latter is also associated with abnormal glucose levels (Lopaschuk and Stanley 1997). Our results show how the three types of cardiomyopathy pathways (in KEGG) and the cardiac muscle contraction pathway are found significantly associated to the traits glucose and waist-to-height ratio. Furthermore, the catalog of published genome-wide association studies (Hindorff *et al.* 2009) reports a meta-analysis in which 17 genes were found significantly associated to fasting glucose (Dupuis *et al.* 2010). Among these reported genes, two genes (ADCY5 and TCF7L2) were also found annotated to ARVC and dilated cardiomyopathy pathways (Ogata *et al.* 1999). We present the variance explained by the SNPs as a preliminary result; because the SNPs chosen for the analysis were those whose *P* values of association were below 0.05, there is an expected degree of variance explained by the subset chosen for the analysis. Subsequently, the present analysis is likely to overestimate the variance explained by the SNPs, and a better methodology needs to be developed. Still, because of the small sample size of the study (n = 719), we do not claim to detect the link between the ARVC pathway and the trait glucose; to confirm this result, replication in another cohort would be required.

The rigorous evaluation of pathway methodologies is very challenging, but the application of permutations can help in their assessment. Our results demonstrate that the circular genomic permutations provide robust associations. A thoughtful consideration of the advantages and disadvantages on the window-size annotation should be done according to the objective of the study. Due to the various possible sources of bias, keeping the annotation window size shorter than 20 kb is recommended. Our new methodology permits the spurious associations that are detected when not accounting for genomic structure to be discarded. According to the hypergeometric-theoretical results, ∼1,400 pathway trait results have a *P* ≤ 0.05; however, empirical results suggest that ∼60% of them could arise just by chance. In this study, we applied Fisher’s combination test and a hypergeometric test to test for pathway significance; however, this permutation approach can be applied to any SNP-to-gene or SNP-to-pathway *P* value estimation methodology and to any other gene sets or pathway resources.

## Supplementary Material

Supporting Information
