# Supplementary material for: Uncovering Networks from Genome-Wide Association Studies via Circular Genomic Permutation
Source: G3 (Bethesda). 2012 Sep 1;2(9):1067–75. doi: 10.1534/g3.112.002618 (PMC3429921; doi:10.1534/g3.112.002618)
Supplement: Supporting Information [file supp_2_9_1067__index.html]

Supporting Information 

# Uncovering Networks from Genome-Wide Association Studies via Circular Genomic Permutation

## Supporting Information for Cabrera *et al.*, 2012

**Files in this Data Supplement:**

- Figure S1 - Hypergeometric-theoretical p-values Vs Hypergeometric-empirical p-values at distance 0 (PDF, 408 KB)
- Table S1 - Trait Descriptions (.xlsx, 41 KB)
- Table S2 - Hypergeometric-empirical Significant Tests Distance 0 (.xlsx, 75 KB)
- Table S3 - Hypergeometric-empirical Significant Tests 20kb (.xlsx, 81 KB)
- Table S4 - Distance 0 and 20kb Hypergeometric-empirical Significant Tests (.xlsx, 36 KB)
