# Supplementary material for: Uncovering Networks from Genome-Wide Association Studies via Circular Genomic Permutation
Source: G3 (Bethesda). 2012 Sep 1;2(9):1067–75. doi: 10.1534/g3.112.002618 (PMC3429921; doi:10.1534/g3.112.002618)
Supplement: Supporting Information [file supp_2.9.1067_FigureS1.pdf]

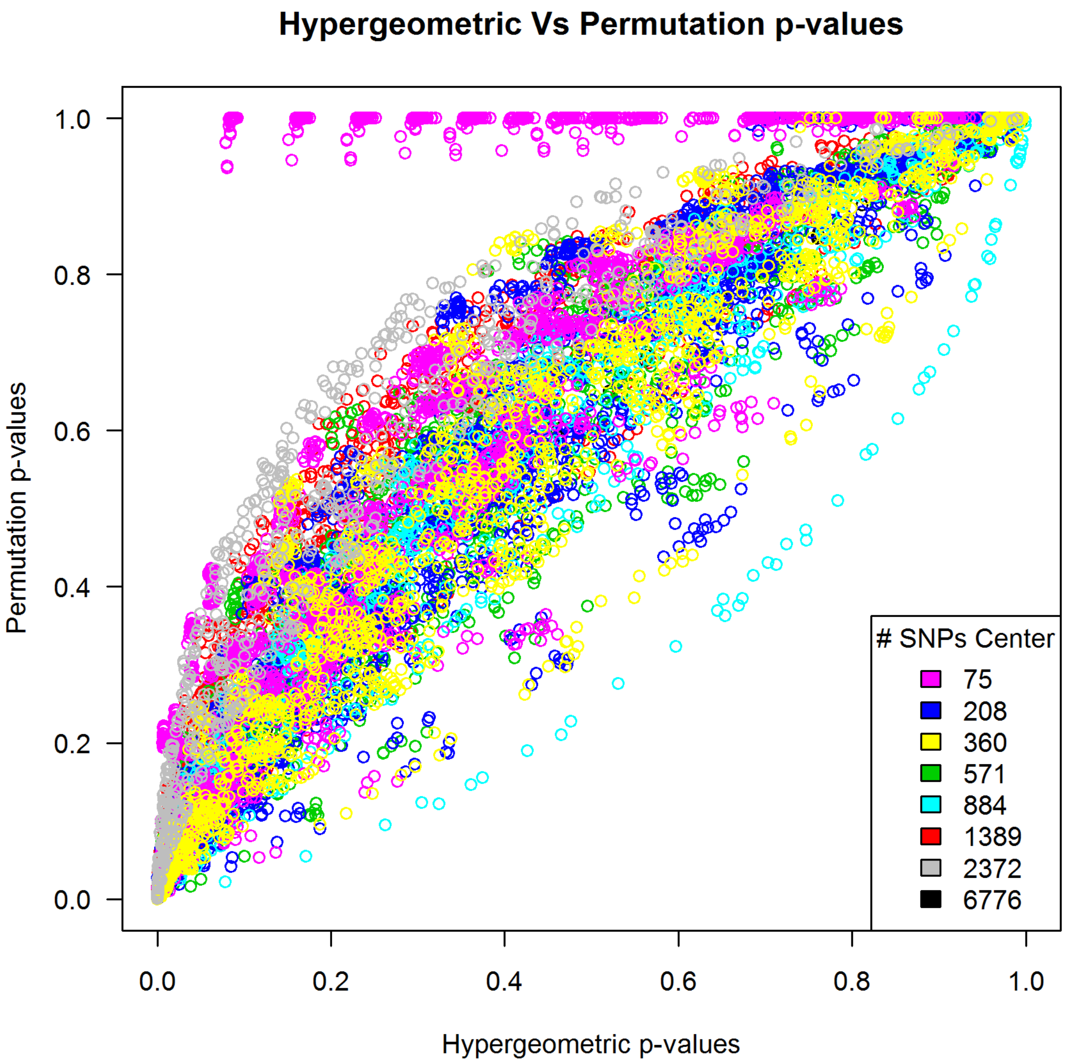

Figure S1 Hypergeometric-theoretical p-values Vs Hypergeometric-empirical p-values at distance 0. Visual representation of the relationship of the hypergeometric-theoretical p-values (x-axis) compared to the hypergeometric-empirical p-values (y-axis). To assess the effect of the size of the pathway, p-values are colored by the total number of SNPs in the pathway. For this representation pathways were clustered using k-means with 8 groups. The legend represents the centers of that size group. The red line represents the trend which would be followed if the hypergeometric-empirical p-values would match perfectly to those of the hypergeometric-theoretical p-values.
